# My English sounds better than yours: Second-language learners perceive their own accent as better than that of their peers

**DOI:** 10.1371/journal.pone.0227643

**Published:** 2020-02-07

**Authors:** Holger Mitterer, Nikola Anna Eger, Eva Reinisch

**Affiliations:** 1 Department of Cognitive Science, Faculty of Media and Knowledge Sciences, University of Malta, Msida, Malta; 2 Institute of Phonetics and Speech Processing, Ludwig-Maximilians-Universität Munich, Munich, Germany; 3 Acoustics Research Institute, Austrian Academy of Sciences, Vienna, Austria; Potsdam University, GERMANY

## Abstract

Second language (L2) learners are often aware of the typical pronunciation errors that speakers of their native language make, yet often persist in making these errors themselves. We hypothesised that L2 learners may perceive their own accent as closer to the target language than the accent of other learners, due to frequent exposure to their own productions. This was tested by recording 24 female native speakers of German producing 60 sentences. The same participants later rated these recordings for accentedness. Importantly, the recordings had been altered to sound male so that participants were unaware of their own productions in the to-be-rated samples. We found evidence supporting our hypothesis: participants rated their own altered voice, which they did not recognize as their own, as being closer to a native speaker than that of other learners. This finding suggests that objective feedback may be crucial in fostering L2 acquisition and reduce fossilization of erroneous patterns.

## Introduction

At one point in time, a language-learning company ran a TV commercial making fun of a German accent in English, with a German coast guard confusing the words “think” and “sink”. Replacing the “th” sound with /s/ is indeed a common mistake in German-accented English [1]. Maybe surprisingly, the commercial tends to work on German audiences as well. This leads to the question why many German learners laugh at such jokes, yet continue making the same mistake themselves.

One obvious possibility would be that the Germans who laugh at such jokes are not the ones who make the mistake. However, there are other possible explanations. Maybe learners have a bias to implicitly overestimate the relative level of their own accent in comparison to others. Such a bias would have far-reaching consequences. It would, first of all, explain the above paradox, because listeners then perceive errors in the speech of others more easily than in their own speech. Moreover, such a bias would have consequences for the perception and improvement of a wide range of aspects of a given accent, including those aspects that are less well-known than the infamous "th" for German learners that is typically used when Germans are portrayed speaking English. For instance, it would also include the prosodic level where German learners need to adjust their speech rhythm to sound more like a native (L1) English speaker, which they only seem able to achieve partially ([2], for another example of prosodic transfer, see [3]). The suggestion that second language (L2) learners perceive their speech as more target-like than it actually is would allow us to help explain this pattern of maintaining an accent even after years of practicing. If L2 learners perceived their own accent as more target-like than it is, their speech monitoring processes would fail to pick up on non-target-like pronunciations in their own speech, hence alleviating the perceived need for further improvement. In this paper, we investigate whether L2 learners indeed perceive their own accent as more target-like than that of their peers.

There is evidence that L2 learners *comprehend* an accent better if that accent is similar to their own. [4] found that L2 learners sometimes comprehend L2-accented speech better if speaker and listener share their first language, even though this effect may be moderated by the proficiency of both the talker and the listener [5]. This reveals an interesting difference between native-language and L2 processing, since, in the L1, not the own voice but a typical voice leads to perception benefits [6].

The study of [4] compared the comprehension of L2 speakers of English by groups of learners from the same versus different language backgrounds (e.g., Korean learners vs. Chinese learners listening to Korean-accented English). A recent study by [7] went a step further and compared comprehension differences within a learners' community. They tested how well German learners can comprehend German-accented English, with the crucial manipulation whether learners were listening to their own voice or someone else’s English. This was tested with minimal word pairs containing phonological contrasts that are typically close to neutralized in German-accented English [7, 8, 9, 10]. [7] showed that most German learners did not produce the contrast as native speakers do, however, they did not neutralize it either. Rather, they produced slightly different targets for these two different vowel categories distinguishing *bet* and *bat* using different acoustic cues—although not as clearly as native speakers. Then, in a second step, they tested with the same group of learners how well these residual cues to the L2 contrasts were utilized in a minimal-pair identification task. That is, participants heard, for instance, recordings of *bet* spoken either by themselves or an unfamiliar L2 speaker and had to decide whether *bet* or *bat* was the intended target. Critically, learners across a range of proficiency groups were successful more often in picking the intended word when hearing their own recordings than when hearing the recordings of others. It is important to note that the minimal-pair identification task supplies an objective measure, that is, the success rate of decoding the intended word. This was crucial since in that experiment participants could recognize their own voices. An objective measure hence avoided biases such as liking (or disliking) one's own voice more than that of others or over- or underestimating one’s own L2 performance.

The results of [7] suggest that learners are better able to *comprehend* their own accents. However, the fact that learners can guess the intended target better for their own production does not necessarily mean that they also assume that their own productions are closer to the native speakers’ phonetic categories. An analogy might clarify this; consider listening to the voice of the cartoon character Daffy Duck, which has an exaggerating lisp. Getting used to and better comprehending this voice does obviously not mean to accept the lisp as being part of the standard pronunciation. Similarly, the higher success rate in the minimal-pair identification task with the own voice compared to others does not necessarily translate into the perception that the own accent is closer to L1 speakers than that of others. However, since perception of novel L2 sounds is shaped by the phonological and phonetic system of the learners' L1 (see, e.g., [11]), perceptual differences of L2 sounds may be more subtle than, for instance, lisp. In addition, previous studies indicated that exposure to accented speech in others does not only help comprehend it in terms of accuracy [12] and processing speed [13, 14] but may also cause listeners to be less harsh in judging the accent [15, 16, 17, 18]. Based on that—in combination with the idea that speakers may be especially frequently exposed to their own productions [7, 19] speculated that better comprehension and higher acceptance of an accent may just be the two sides of a coin. In the current paper, we test this possibility explicitly by asking L2 learners to rate the accent of their own voice compared to that of other learners.

At this stage, it is worth considering whether it is in fact useful to ask L2 learners to judge the accentedness of their own speech, because it has been repeatedly pointed out that the goal of an L2 learner should be intelligibility—which is defined as the addressee’s comprehension of the intended words, similar to the word-error rate metric used in automatic speech recognition—rather than imitating a native speaker and that these two goals are not necessarily the same [20, 21]. First of all, there is not “the native speaker” that may be shared as a goal by all learners. All languages (especially English) show considerable variation across varieties, and it is difficult to ascertain what a given learner may view as a target. Moreover, it has been demonstrated that while accented pronunciation often decreases intelligibility (e.g., [22, 23]), this does not necessarily mean that accented speech is less intelligible in all cases [21, 24]. In sum, although there is a relation between foreign accent, intelligibility, and comprehensibility—which is defined as the perceived ease of comprehension—even a speaker with a strong accent may be fully intelligible, and understanding may not necessarily be effortful (see also [25]).

In the context of the current question, however, it is nevertheless useful to ask learners about accentedness. First of all, intelligibility measures can suffer from ceiling issues when speech is presented in absolute quiet, and such measures may underestimate the impact of accentedness on the cognitive effort required to comprehend a speaker. Secondly, when a learner monitors him- or herself, intelligibility is not available as a category, since the speaker knows what message he or she produces, so that even an—to a third party—unintelligible message will be restored for the speaker producing the message [26]. Therefore, it is useful to pose the question how accented speakers sound to themselves.

However, the prospect of asking learners to rate the accentedness of their own versus others' voices raises another immediate concern: the questionable validity of accent ratings when one judges oneself. This was, in fact, one of the motivations for [7] to use a task in which success and failure could be determined objectively. With a free rating task, both “self-serving” overestimation as well as underestimation driven by modesty are conceivable. That is, rating one’s own accent is riddled with potential biases [27]. We circumvented this problem by recording participants, and then altering the voice characteristics of these recordings before asking for accent ratings. More specifically, we used only female participants and altered their recordings so that their voices sounded more like a male voice (see Method for details). This manipulation preserved all aspects of the accent—segments and prosody alike—while rendering the voice unfamiliar. Our prediction was that participants rate their own altered voice as better than that of others, even if—due to the voice alteration—they are not aware that they are also rating their own voice.

If learners indeed consider their own accent as more target-like than that of their peers, it would have theoretical and practical consequences. First, it would highlight the need for external feedback in L2 acquisition, since learners would standardly assume that they are closer to the target than they are. External feedback would therefore be necessary to break this pattern. Second, it would explain why German learners of English hear a German accent in their peers even if they persist in making similar errors themselves. Third and finally, this would also contribute to explaining so-called *fossilization* in L2 acquisition, which describes the fact that L2 learners at some point typically do not further progress towards the L1 target [8, 28]. That is, if learners are able to distinguish the categories of the L2 reasonably well and, in addition perceive their own accent as relatively good in comparison to other learners, there would be little motivation to improve their pronunciation.

## Materials and methods

### Participants

Twenty-four female students or former students of the University of Munich participated for pay. They all were native speakers of German and reported no history of speech, language, or hearing problems. The demographic details of the participants are presented in Table 1. None of them had lived in an English-speaking country for longer than 6 months. The participants were recruited at the University of Munich (LMU) in October 2018 where the research took place. The study was part of an agency-funded research proposal (see acknowledgments) which required no separate ethics approval. All participants gave written informed consent and the study was conducted in accordance with the Declaration of Helsinki. While the sample is not representative of university students, there was a wide range of degrees that the participants read for, ranging from physics to theatre (17 different degrees in the sample). Participants were recruited through flyers and the existing database of participants that participated in earlier studies of the Institute of Phonetics at the LMU. The sample size followed the study by [7], who also studied perception of foreign accent of self vs. other. They found a highly reliable effect, and we used their effect size to establish power for by-item and by-participant tests of the self-other effect, leading to values of 0.84 by participants and 0.99 by items.

**Table 1:** Demographic details of the participants.

	Mean	Standard Deviation	Range
Age	22.3	4.1	18–30
Starting Age for the Acquisition of English	9.4	1.9	4–12
Duration of schooling in English	8.7	1.8	5–14
Self-rated proficiency in English			
Hearing	2.33	1.25	1–5
Reading	2.50	1.00	1–5
Writing	3.13	0.88	2–5
Speaking	3.00	1.02	1–5
Overall	2.88	0.93	1–5

The scale of the self-evaluation is 1–7, with 1 being the best score.

### Materials and stimuli

We used the 60 English sentences used by [4] in their study of accent perception. The sentences are syntactically simple and contain words that are likely familiar to non-natives. Naturally, they contain sounds that have been shown to be difficult to produce for German leaners, for instance, word-final voiced stops and fricatives, the open-mid front vowel /æ/, the contrast /w-v/, light vs. dark /l/, and the dental fricatives /θ/ and /ð/.

At this stage, it is important to note that we do not mean to imply that a non-native accent can be reduced to confusable phonemes; clearly, slight mismatches between L1 and L2 categories, even if transcribed with the same IPA symbol, and prosodic features also contribute to an L2 accent. All these characteristics were retained in the changed voices, hence listeners were confronted with a global accent.

To generate stimuli for the rating task, the 24 participants were recorded reading the 60 sentences twice in a sound-proof booth using a diaphragm microphone (Neumann Microphone, type TLM 103). The order of sentences was randomized individually for each participant and all words were presented once before repeated. Sentences were presented one by one on a computer screen using Speechrecorder software [29] which allows to instantly re-record bad or hesitant recordings of a given sentence until a fluent rendition is recorded. For each speaker, one utterance per sentence was selected for further processing. This was by default the first utterance, unless there were lip-smacking sounds or hesitations.

The recordings were then altered using the Change-Gender function in Praat (v 6.0.14; [30]). To change a female voice into a typical male voice, both f0 and the formant frequencies need to be lowered. To achieve this, the f0 of the original stimuli was multiplied on average by a factor of 0.59 and the formants multiplied on average by a factor of 0.82. These factors were, however, fine-tuned separately for each speaker, based on their voice characteristics, leading to range of f0 (multiplication factors .51-.76) and formant shifts (multiplication factors .79-.85). These variations were based on the original value of the speaker, that is, a speaker who already had a relatively low f0 or formant pattern was shifted less than a speaker with a very high f0 and rather high formants. Note that the two dimensions are to some extend independent [31], that is, an above average correction for f0 may go hand in hand with a sub-average adjustment for the formants.

### Procedure

All participants took part in two sessions: one for the recordings (described above), and one for the perception experiment, which was divided into the main rating task and a brief control experiment. At the start of the first session, participants signed an informed consent form. After the recording session, they filled in a language background questionnaire with special focus on their history of learning English. Between four and eight weeks (mean 6.2) after they were recorded, participants returned for the main session in which they judged the accent of the altered voices. They were informed about the procedure by means of written instructions in English.

For the perception experiment, participants were seated in a sound-proof cabin in front of a laptop computer. On each trial, participants saw the question “how well is the sentence pronounced?” and a six-point scale from 1 “well” on the left to 6 “poorly” on the right on the computer screen. This rating scheme follows the German marking system at school which goes from 1 (very good) to 6 (deficient). Note that by asking how well the sentence was pronounced instead of asking how accented it was (see, e.g., [32] [17]), we put our question about the accentedness of the production in a positive way (i.e., “how good” rather than “how bad/accented”) and aimed at triggering learners to compare the pronunciation to a model of how they thought the sentence should sound. One second after the appearance of the rating scale, the auditory stimulus was presented over headphones at a comfortable listening level. Responses were logged by pressing the number buttons on the keyboard and were allowed only from 1.5 seconds after the sound started playing. This was to ensure that participants did not respond before the end of the sentence. Trials were separated by a 500ms inter-trial-interval (ISI). Participants heard the sixty sentences from their own and three other altered voices (see Design for how these voices were selected), leading to 240 trials presented in a random order. Every 60 trials, participants were allowed to take a self-paced break. The experiment was implemented in Psychopy2 (Version 1.83.01; [33]), and took approximately 40 minutes to complete.

After this main rating task, a second task was administered to assess whether, despite the changed-gender manipulation, participants would recognize the similarity of their altered voice to their own actual voice. Each participant was presented with ten of the sentences produced either by themselves or the other three speakers. The subset of these ten sentences was the same for all participants. Participants were asked to respond to the question “how much does this sound like you?” on a scale from 1 to 6. This time, 1 indicated “a lot” and 6 indicated “not at all”. Again, responses were only allowed from 1.5s after sentence onset and the ISI was 500ms. Finally, after this task, participants were asked explicitly by the experimenter whether they had recognized one of the voices as their own voice.

### Design

In order to ensure that during the main experiment each participant would hear a similar range of good vs. poor accents, all recordings were first ranked with regard to their accentedness. Then six groups of four participants were created, so that each group contained one speaker from each quartile.

The ranking was based on accent ratings for each sentence on a 9-point scale. Raters were three phonetically-trained advanced German learners of English who use English on a daily basis. In addition, a native speaker of British English rated a subset of the sentences for validation. Ratings were blocked by sentence, that is, each rater heard a given sentence from all speakers before moving on to the next sentence. Within each block, the order of speakers was randomized. Raters first heard two sentences from each speaker as a warm-up to familiarize themselves with the range of accents. Each of the German raters judged all speakers for twenty sentences, and the British speaker rated all speakers on the ten sentences that were most diagnostic for the overall score in the ratings of the three German raters, that is, those that had the highest correlation between the mean rating for a given sentence and the mean rating of the other 59 sentences. Over raters, ratings were highly correlated both amongst the three German raters (r_s_ = 0.79–0.82) as well as between the German raters and the British-English rater (r_s_ = 0.73–0.82).

Based on these ratings, we generated six groups of four participants containing one participant from each quartile. Each participant would hence hear herself and three other speakers from the other quartiles. With this design, each participant heard her own altered voice on 25% of the trials and others' voices on the remaining 75%.

### Analysis

All statistical analyses presented are based on linear mixed-effects models conducted in R (v3.2.5) using the package lmerTest (v.3.0.1, [34]). We used a normal, rather than a generalized, linear mixed-effects model, that is, the model predicts the observed values, rather than some transform of them. While there has been an active debate whether data from a rating scale may be analysed with such a normal linear model, this is generally deemed acceptable [35].

The linear mixed-effects model produces t-tests for each experimental effect, for which degrees of freedom (and hence p-values) were estimated based on the Satterthwaite's method as implemented in the R library lmerTest. Random effects were used for participant and item (i.e., the sentence played on a given trial). The random-effects structure was near maximal, that is random slopes were used for fixed effects that varied over participants and items, except for the exclusion of correlation parameters between random effects [36]. As an additional control variable, a random intercept for voice source was added, that is, which speaker’s voice was presented on a given trial. This random effect captures overall differences in accent quality between participants. The data and analysis scripts are available at https://osf.io/7tg8p/.

## Results

**Fig 1:**
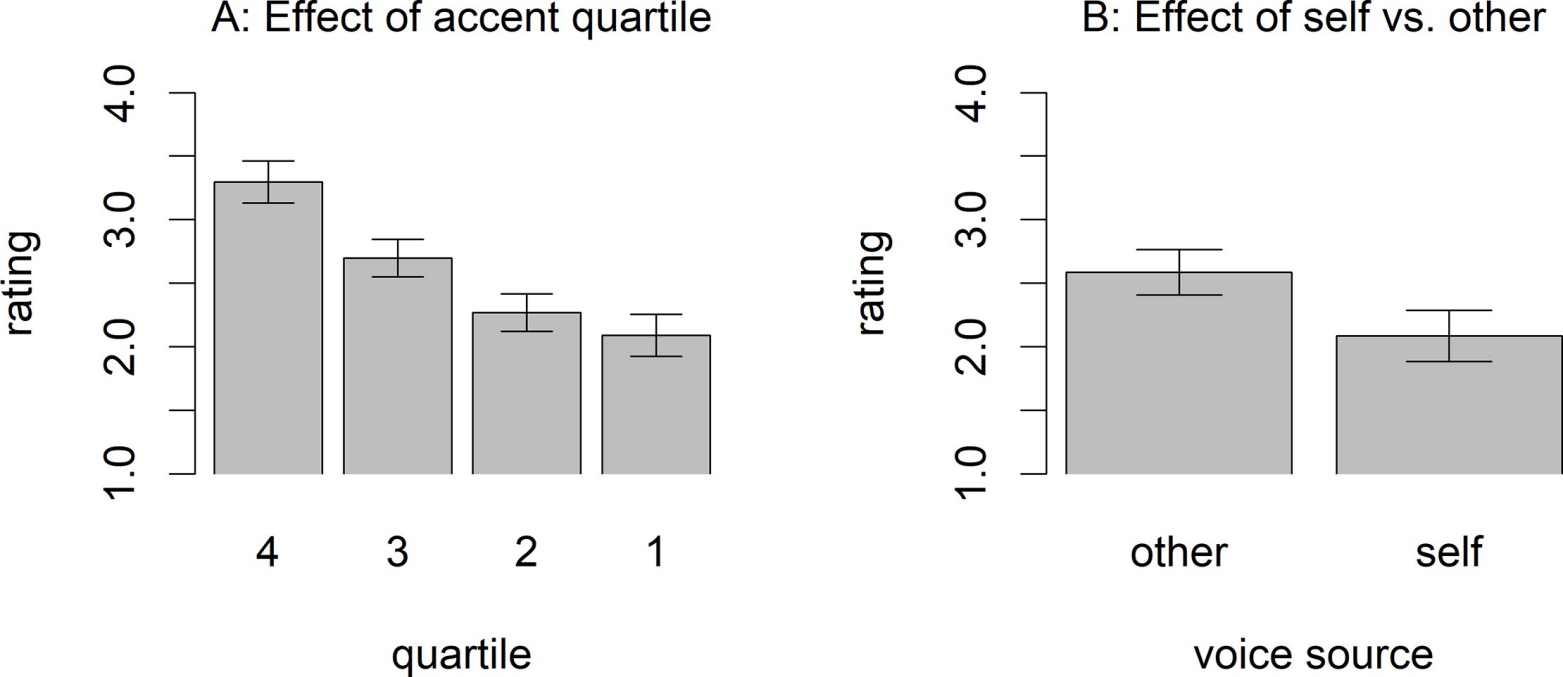
Results from the accent-rating task. Within the response options (i.e., "rating" plotted on the y-axis), 1 indicates “very good pronunciation” and 6 “poor pronunciation”. Here, means are shown only for a range from 1 to 4. The left panel shows the result for the rating of others' voices only, depending on their quartile, showing that higher quartiles got better ratings. The right panel shows the effect of voice source, with better ratings for own-voice trials. Error bars show standard errors estimated with the R package emmeans [37].

Our main question was whether accent ratings were different for trials in which participants judged their own altered voice compared to those of other speakers. However, first we wanted to investigate whether participants really did judge accentedness. Given the potential influence of hearing one's own (altered) voice, we used only trials in which others' voices were judged and tested whether the “source quartile” (based on our initial ratings) had an influence on participant ratings. This was clearly the case: average ratings decreased monotonically with quartile (see Fig 1, left panel). In a linear mixed-effects model with quartile as the only fixed effect (contrast coded as 1.5, .5, -.5, -1.5, for the quartiles 1 to 4, respectively), this effect was significant (b = 0.-46, t(24) = -8.54, p < 0.001). Looking at the patterns produced by individual participants, we calculated the mean rating for the three other learners that a given participant rated and rank-ordered these based on the ratings. The ratings of 18 out of the 24 participants re-produced the rank orders from the quartile assignments, that is, they rated the accent from the participant with the highest quartile highest, the one from the middle quartile second, and the participant from the lowest quartile lowest (i.e., 3–2–1). Four participants switched the rank order on the two lower-ranked participants (i.e., 2–3–1), and two switched the two higher ranked participants (i.e., 3–1–2). However, no participant produced a rank order that was completely dissociated from the quartile assignments based on the phonetician’s rating. This indicates that participants were engaging in the task of judging how well the sentence they heard was pronounced.

This leads to our main question whether ratings for learners' own voice were better than ratings for other voices. This was clearly the case: the mean rating for own-voice trials was 2.09 (close to the mark “good” in the German marking system) and that for other-voice trials was 2.59 (between the marks “good” and “satisfactory”, see the right panel of Fig 1). A linear mixed-effects model with accent rating as the dependent variable and voice as independent variable (contrast coded as self = -0.5 and other = 0.5) showed that this effect was significant (b = 0.50, t(22) = 5.30, *p* <0.001). It is worthwhile to note that the effect of hearing one's own versus another voice is similar in size to the effect of quartile, both being around one half. This means that, hearing oneself, the perceived accentedness is one quartile higher. Hence, an L2 learner who is average (50^th^ percentile) would perceive herself as being better than three out of four other speakers (i.e., 75^th^ percentile).

Results of the voice-similarity judgement task that followed the main accent rating task showed that participants rated their own altered voice as more like their own voice than that of others. On the scale from one to six, participants rated, on average, the similarity of the presented voice to their own voice as 2.00 (equivalent to “good” in the German marking system) when they heard their own altered voice but only as 3.25 (close to 3, categorized as “satisfactory” in the German marking system) when hearing another altered voice. A linear mixed-effects model with similarity rating as dependent variable and voice (self vs. other) as independent variable showed that this difference was significant (b = 1.25, t(23) = 6.28, *p* <0.001).

During debriefing only three participants indicated to have recognized their own (altered) voice. While one participant clearly showed evidence of explicit recognition with high similarity ratings for the own (mean: 1.3) but very low ratings for the other voices (mean: 5.4), another of these three participants thought that *all* sentences played in the similarity rating task were from her own voice, giving high similarity ratings to her own and others' voices (mean = 1.0 for both cases). The third one of these three participants was in between these patterns with slightly lower similarity ratings for the own voice (mean: 1.5, note that a low mark means a high-perceived similarity) and relatively low ratings for other voices (mean: 3.7). Notably, the effect of voice (self-other) described above is virtually unchanged, if we exclude the three participants who did recognize their own voice (b = 0.50, t(19) = 4.97, p <0.001)

An additional model tested to what extent the perceived similarity of the altered voice to one's own voice influenced accent ratings. From the similarity ratings, we generated the average similarity rating for each pair of rater and listener (that is, how similar to her own voice a given participant rated the four voices in her stimulus set including their own). Since the pairings were the same for the accent and similarity rating tasks, this average similarity rating could be used as a co-variate in the analysis of the accent ratings. That is, for all trials on which participant X rated participant Y, the average similarity rating by participant X for participant Y was used. Because these are collinear with the fixed effect of Voice (i.e., self vs. other), the similarity ratings were normalized. That is, for “other” similarity judgements, the mean of all “other” judgments was subtracted, and for “self” judgements, the mean of all “self” judgements was subtracted. This measure of perceived similarity then reflects, for the level own-voice, whether a given participant rated her own altered voice as more similar to her real voice than the other participants. For the other-voice level, it indicates whether a given other voice was perceived as more or less different than the average other voice. A linear mixed-effects model was run with accent rating as the dependent variable and Voice (self vs. other), perceived Similarity (as a numeric predictor) and their interaction as fixed factors. The categorical variable Voice was dummy coded with the level “self” mapped on the intercept. The results showed an effect of Voice (b = 0.50, t(21) = 5.60, *p* < 0.001), no significant effect of perceived Similarity on the “own-voice” intercept level of the Voice factor (b = -0.07, t(41) = -0.58, *p* = 0.56) but a significant interaction (b = 0.34, t(48) = 2.56, *p* = .014). This suggests that, when rating the own altered voice, participants did not give better scores if they perceived the voice as more similar. However, other voices were rated better when they were also perceived as above-average similar to the own voice.

## Discussion

This experiment set out to test whether L2 learners would rate their own L2 productions as better, that is, less accented, than productions of fellow learners from the same language and social background. Such a bias would have consequences for their ability to further improve their L2 pronunciation, as they perceive themselves as more target-like than they are. Note that by asking how well a given sentence was produced we obtained accentedness ratings by asking learners to compare a given stimulus to how they considered a native speaker to sound. We used a measure of accentedness rather than intelligibility since in a situation of self-monitoring learners know what they intend to say, hence are fully intelligible to themselves. Critically, we strove to ask L2 learners to rate their own accent in comparison to other learners without them noticing that they are doing so. By changing the voice characteristics, we were largely successful in preventing learners from recognizing their own voice: only three out of 24 participants claimed to do so, and the explicit recognition partly dissociated from similarity ratings that those participants gave in a similarity-rating task. Moreover, we were able to show that participants focussed on rating the accentedness of the voices, given that their ratings largely followed the assignment of learners into quartiles, which had been determined based on phoneticians’ and a native-speaker’s ratings. This shows that participants were, first, focussing on accentedness and, second, were not fully aware that they are rating their own voice in comparison to others.

Nevertheless, learners rated their own accents as better than that of others. Note that this cannot be due to real differences in accentedness, as this comparison is focussing on the *same* stimuli rated by different learners. That is, the identical stimulus was rated differently (in comparison to others) depending on whether the learner had produced it herself or not. This finding reveals a cognitive bias that may have serious repercussions for L2 acquisition. If learners perceive their accent as better than that of others, this might make them less likely to notice errors. If they fail to notice their errors, continuing this rationale, they are unlikely to correct them as there may be little motivation to further improve. Given that the bias is also relatively large (i.e., one quantile overestimation), this is a realistic scenario, above and beyond just being a statistically significant effect.

There are at least two possible explanations for the higher self-ratings. It may be another instance of the mere-exposure effect [38], which describes that repeated exposure to a stimulus makes it more likeable. Hearing our own voice whenever we speak in the L2 may hence make us “like” that accent, and other, similar accents, more than they would objectively deserve. Alternatively, the comprehension advantage for the own voice [7] may make it easier to comprehend the own voice, which leads to a better rating. Note that these explanations are not mutually exclusive in an L2 learning context.

Rather, both are in line with studies demonstrating that listeners who are frequently exposed to non-canonical speech of others adapt to it. This adaptation is reflected in better understanding and processing on one hand (e.g., [13, 12]), but also in higher acceptance and less harsh judgements on the other [15]. This is in line with other work suggesting that we can quickly adapt to unusual pronunciations in L1 [39, 40] and even in L2 listening [41]. Affective explanations of the mere-exposure effect focus on the idea that unknown stimuli may generally invoke fear (for a review, see [42]). While this may sometimes seem far-fetched for studies that simply show relatively simple stimuli of (invented) commercial products on a screen, it probably rings a bell for any L2 learner. Every new sentence in an L2 bears the danger of not being understood, so being able to process an accent relatively easily may directly influence the appraisal of this stimulus.

Our data may also contribute to explain the finding of *fossilization* in L2 learning. If learners perceive their own accent as better than that of others, they may not feel a need to further improve. This does not imply that learners cannot improve their pronunciation at all. We deem it likely that more practice typically leads to better pronunciation over time, even though learners perceive themselves in a biased fashion. However, perceiving one’s accent as better than it objectively is may be one reason why learners persist in speaking with an accent even after many years of practice, which we refer to as *fossilization*. Crucially, this also means that learners may not profit from pronunciation training that requires them to notice differences between their own speech and that of a native speaker themselves—both in terms of phonemic errors and subphonemic and prosodic detail—without external corrections. That is, our results suggest that external feedback may be essential for pronunciation training to highlight those aspects of the accent that should be improved. Feedback may be provided by teachers or, less costly but not yet fully accurate, computer-assisted learning programs [43]. Interestingly, research on computer-assisted training suggested that learners benefit more from listening to their own, through speech synthesis corrected, productions than from a native model (e.g., [44, 45]). A potential reason for this may be that the mental target model they have developed is also shaped by their own accent, so that a comparison with another speaker does not help detecting errors and accented pronunciation. Providing a more native or more target-like model in their own voice, however, may enable them better to change their pronunciation (see also [46]). While it remains to be shown how external feedback affects one's own accent ratings or how feedback in one’s own voice could be practically integrated in real classroom situations, relying on self-monitoring alone may not be enough to lead to improvement in the L2.
